# microRNA-214-3p Suppresses Ankylosing Spondylitis Fibroblast Osteogenesis *via* BMP–TGF*β* Axis and BMP2

**DOI:** 10.3389/fendo.2020.609753

**Published:** 2021-04-15

**Authors:** Lixiang Ding, Yukun Yin, Yu Hou, Haoran Jiang, Ji Zhang, Zhong Dai, Genai Zhang

**Affiliations:** ^1^ Department of Spine, Beijing Shijitan Hospital, Capital Medical University, Beijing, China; ^2^ Department of Traditional Chinese Medicine, National Cancer Center/National Clinical Research Center for Cancer/Cancer Hospital, Chinese Academy of Medical Sciences and Peking Union Medical College, Beijing, China; ^3^ Department of General Medicine, Huanxing Cancer Hospital, Beijing, China

**Keywords:** ankylosing spondylitis, microRNA-214-3p, osteogenic differentiation, bone morphogenic protein 2, BMP–TGF*β* signaling pathway

## Abstract

Recent investigations suggest microRNAs (miRs) exert functions in fibroblast osteogenesis in ankylosing spondylitis (AS), an inflammatory rheumatic disease. But the mechanism of miR-214-3p in osteogenic differentiation in AS is not clearly understood yet. In this study, fibroblasts were obtained from the capsular ligament of patients with AS and femoral neck fracture and cultured for osteogenic induction and identified. The roles of miR-214-3p and bone morphogenic protein 2 (BMP2) in AS fibroblast osteogenesis were assessed *via* gain- and loss-of-function, alizarin red S staining, and alkaline phosphatase (ALP) detection. Levels of miR-214-3p, BMP2, osteogenic differentiation-related proteins, and BMP–TGF*β* axis-related proteins were further measured. Consequently, miR-214-3p was downregulated in AS fibroblasts, with enhanced ALP activity and calcium nodules, which were reversed by miR-214-3p overexpression. BMP2 was a target gene of miR-214-3p and promoted AS fibroblast osteogenesis by activating BMP–TGF*β* axis, while miR-214-3p inhibited AS fibroblast osteogenesis by targeting BMP2. Together, miR-214-3p could prevent AS fibroblast osteogenic differentiation by targeting BMP2 and blocking BMP–TGF*β* axis. This study may offer a novel insight for AS treatment.

## Introduction

As a highly heritable and prototypic spondyloarthropathy, ankylosing spondylitis (AS) is closely associated with inflammatory response, progressive rigidity, and peripheral arthritis and mainly affects the spine and pelvis (1, 2). AS begins at the early stage of life and always results in physical dysfunction and reduced health-related quality of life (3). With AS progression, chronic inflammation and new bone formation persist in all segments of the spine, and the calcification of vertebral body and paravertebral ligament will aggravate (4). New bone formation is based on increased differentiation of osteoblasts (5). AS patients are at a high risk of spinal fracture, spinal cord injury, osteoporosis, hypertension, cardiovascular diseases, pulmonary complication, and metabolic syndrome (6, 7). As is reported, current anti-rheumatic drug therapies improve back pain, peripheral arthritis acute phase responses, disturbed sleep, and overall quality of life; however, the major contributing factor of AS—new bone formation—is not affected (5). In light of this, the search for novel targets for AS treatment should focus on osteogenic differentiation to prevent new bone formation.

microRNAs (miRs) are small non-coding RNA molecules, involved in many biological processes with interaction with their mRNAs (8). Aberrant miR expression is related to the pathogenesis of rheumatoid arthritis and osteoarthritis (9). A recent study points out that miR-214 is consistently decreased in AS and potentially serves as a non-invasive biomarker for AS diagnosis (10). Importantly, miR-214-3p is involved in osteogenesis of maxillary sinus membrane stem cells (MSMSCs) (11). In this study, we found miR-214-3p could target bone morphogenetic protein 2 (BMP2). BMPs, a subset of the transforming growth factor *β* (TGF*β*) superfamily, exert their roles in bone formation *via* BMP receptors (BMPRs), which in turn activate Smad1/5/8 axis and facilitate osteoblastic gene transcription (12). In particular, high localized expression of BMP-2 at the site of ankylosing enthesitis is involved in ankylosis development in a mouse model of spondyloarthritis (13). Importantly, Shea Carter et al. put forward the notion that activation of BMP pathways may be a very early or even initial event in AS (14). A former research has demonstrated that TGF*β*1 is involved in AS development and is elevated upon complement attack in osteoblasts and osteoclasts (15). TGF*β*1 and BMP2 have significant effects on adhesion, proliferation, and differentiation of primary human osteoblasts (16). It is recognized that the TGF*β*–BMP axis is essential for osteogenesis and bone formation in mammalian development (17). From all of the above, it is reasonable to hypothesize that there may be an interaction among miR-214-3p, BMP2, and TGF*β*-BMP signaling pathway in AS. Thus, a series of experiments were performed in this study to justify the hypothesis.

## Methods

### Cell Culture and Osteogenic Induction

Twenty patients with AS involving both hips and requiring joint replacement from January 2017 to January 2019 were selected as the experimental group (16 males and four females), with an average age of 32.8 ± 1.8 years. These patients met the modified New York criteria (1984) for AS (Raychaudhuri and Deodhar, 2014), free of other immune diseases such as wind dampness. Eighteen patients with femoral neck fracture (free of AS and other immune diseases) who needed open surgery or joint replacement were selected as the control group (12 males and six females), with an average age of 40.7 ± 2.4 years. The specimen was the tissue of the hip joint capsule, which was the waste during the surgery. The tissue of the cut hip joint capsule ligament was collected as the specimen. The ligaments were cut into 0.5 mm^3^ pieces and washed twice with phosphate buffered saline (PBS). Then the ligament pieces were preserved in Dulbecco’s modified Eagle medium (DMEM) (Thermo fisher, Carlsbad, California, USA) containing 10% fetal bovine serum (FBS), 100 U/ml penicillin and 100 μg/ml streptomycin in a 5% CO_2_ incubator at 37°C. After cell adhesion, the ligament pieces were discarded, and the culture medium was changed every 3 days. Cells were separated at 1:3, and cells in the third passage were used for further study. When fibroblast confluence reached 80%, osteogenic differentiation was induced by adding 0.1 μl/L dexamethasone + 10 mmol/L *β*-glycerophosphate + 50 μl/L ascorbic acid (18).

## Hematoxylin and Eosin Staining

The third passage cells were washed with precooled PBS for 10 min, and fixed with 4% paraformaldehyde for 30 min. According to the standard scheme, cells were stained with HE (Beijing Solarbio Science & Technology Co., Ltd, Beijing, China), and observed under the optical inverted microscope (Leica, Solms, Germany).

### Immunocytochemistry

Cleaned, acidic, and sterilized cover slides were placed in a cell culture dish, added with cell suspension and cultured for 24–48 h. When the cover slides were paved with cells, the culture medium was removed, and the slides were washed in PBS, fixed with 4% formaldehyde for 15 min, and incubated with 0.3% Triton X-100 for 20 min. After PBS washing, cells were blocked for 30 min with 1% bovine serum albumin (BSA), and incubated for 1 h with primary antibody anti-Vimentin (1: 100, ab8978, Abcam, Cambridge, MA, USA) at 37°C. Subsequently, cells were incubated for 30 min with goat anti-mouse IgG H&L (HRP) (1:2000, ab205719, Abcam). Finally, cells were conventionally stained with 2, 4-diaminobutyric acid (DAB) and counterstained in hematoxylin. The positive expression of vimentin in fibroblasts was visualized under a light microscope at 40× magnification.

## Cell Transfection and Grouping

BMP2 cDNA was cloned into pcDNA3.1 vector (Invitrogen Inc., Carlsbad, CA, USA). BMP2 small interfere (si) RNA (si-BMP2) was designed and synthesized by Genepharma Co., Ltd. (Shanghai, China) to silence BMP2 expression. Meanwhile, miR-214-3p mimic, miR-214-3p inhibitor, and miR-negative control (NC) were designed and synthesized by Thermo Fisher Inc. (Shanghai, China). Fibroblasts were transfected using Lipofectamine™ 2000 reagent (Invitrogen Inc., Carlsbad, CA, USA). The transfection efficiency was verified using RT-qPCR.

### Reverse Transcription Quantitative Polymerase Chain Reaction

The one-step method of TRIzol (Invitrogen, Carlsbad, CA, USA) was employed to extract total RNA, and the extracted high-quality RNA was confirmed using ultraviolet analysis and formaldehyde denaturation electrophoresis. RT-qPCR was conducted based on instructions of RT-qPCR kit (Thermo Fisher Scientific, Shanghai, China) with glyceraldehyde-3-phosphate dehydrogenase (GAPDH) as an internal reference for BMP2 and U6 for miR-214-3p. PCR primers were provided by Shanghai Sangon Biotechnology Co., Ltd. (Shanghai, China) (**Table 1**). The amplification and dissolution curves were confirmed after the reaction, and data were analyzed by 2^–ΔΔCt^ method (19).

**Table 1:** Primer sequence of RT-qPCR.

Gene	Primer sequence
BMP2	F: 5′‐GTCGACCATGGTGGCCGGGACC‐3′
	R: 5′‐TGCTGTACTAGCGACACCCACA‐3′
GAPDH	F: 5′-GGGAGCCAAAAGGGTCAT‐3′
	R: 5′-GAGTCCTTCCACGATACCAA‐3′
miR-214-3p	F: 5′-ACAGCAGGCACAGACAGGCAGT‐3′
	R: 5′-ACTGCCTGTCTGTGCCTGCTGT‐3′
U6	F: 5′-CGCTTCGGCAGCACATATAC‐3′
	R: 5′‐AATATGGAACGCTTCACGA‐3′

RT-qPCR, reverse transcription quantitative polymerase chain reaction; BMP2, bone morphogenic protein 2; GAPDH, glyceraldehyde-3-phosphate dehydrogenase; miR-214-3p, microRNA-214-3p.

### Alizarin Red S Staining and Quantification

At 21 days after osteogenesis induction, fibroblasts were fixed in 4% paraformaldehyde and stained with 1% ARS (pH 4.3) for 15 min (12). After being washed three times, the cells were observed under a microscope and photographed. The stained cells were decolored with 10% cetylpyridinium chloride monohydrate (Sigma-Aldrich, Merck KGaA, Darmstadt, Germany). A 200 µl aliquot was transferred to a 96-well plate, and the absorbance was determined at 562 nm.

### Alkaline Phosphatase Detection

ALP activity of fibroblasts was detected as per the manufacturer’s instructions (Beijing Solarbio Science & Technology Co., Ltd, Beijing, China). At 7, 14, and 21 days after osteogenesis induction, cells were collected and lysed and added with 100 μl reaction substrates. The termination solution was added to the cells after reacting at 37°C for 15 min. After that, the absorbance at 405 nm was determined by a microplate reader to calculate ALP activity.

### Dual-Luciferase Reporter Gene Assay

The database (http://starbase.sysu.edu.cn/) (20) was used to predict the target binding site of miR-214-3p with BMP2. BMP2 fragment containing the binding site of miR-214-3p was cloned into the pmirGLO oligosaccharide enzyme vector (Promega, Madison, WI, USA), and the pmirGLO-BMP2-wild type (Wt) reporting vector was constructed. The pmirGLO-BMP2-mutant type (Mut) was constructed with the mutant binding site of miR-214-3p based on pmirGLO-BMP2-Wt. After that, the constructed vectors were transfected into fibroblasts and then co-transfected with miR-214-3p mimic and miR-NC respectively. After 48 h, the luciferase activity was assessed using dual luciferase reporter gene assay system (Promega, Madison, WI, USA), and the relative activity was calculated as the ratio of firefly luciferase activity to renilla luciferase activity.

### Western Blot Analysis

The proteins were extracted to determine the concentration as per the instructions of bicinchoninic acid kit (Thermo Scientific Pierce, Rockford, IL, USA). The extracted proteins were boiled and run on sodium dodecyl sulfate polyacrylamide gel electrophoresis from 80 V to 120 V. Afterward, the proteins were transformed into the polyvinylidene fluoride membranes (Millipore Corp., Billerica, MA, USA). The membrane was blocked and incubated with primary antibodies (Abcam Inc., Cambridge, MA, USA) at 4°C overnight: BMP2 (1:1,000, ab14933), BMPR2 (1:1,000, ab96826), Smad5 (1:1,000, ab40771), GAPDH (1:1,000, ab8245), Collagen I (COLI) (1:1,000, ab96723), osteocalcin (OCN) (1:500, ab93876), runt-related gene 2 (Runx2) (1:1,000, ab76956) and p-Smad5 (1:500, ab76296). Then the membrane was rinsed in tris-buffered saline tween (TBST) and cultured with secondary antibody (Santa Cruz Biotechnology, Inc, Santa Cruz, CA, USA) labeled by horseradish peroxidase. After TBST washing, proteins were visualized by enhanced chemiluminescence reagent and developed by Gel EZ imager (Bio-Rad Laboratories, CA, USA). Finally, the target band was analyzed with Image J software (National Institutes of Health, Bethesda, Maryland, USA) for gray value analysis.

### Statistical Analysis

SPSS 21.0 (IBM Corp., Armonk, NY, USA) was employed for data analysis. Kolmogorov–Smirnov test showed whether the data were in normal distribution. The measurement data were exhibited as mean ± standard deviation. Comparisons between two groups were analyzed with *t* test; among multiple groups were assessed with one-way analysis of variance (ANOVA) or two-way ANOVA, and pairwise comparisons after ANOVA were conducted by Tukey’s multiple comparisons test. *p* value was obtained by two-tailed test and *p* < 0.05 inferred a statistical difference.

## Results

### Isolation and Identification of Fibroblasts

**Figure 1:**
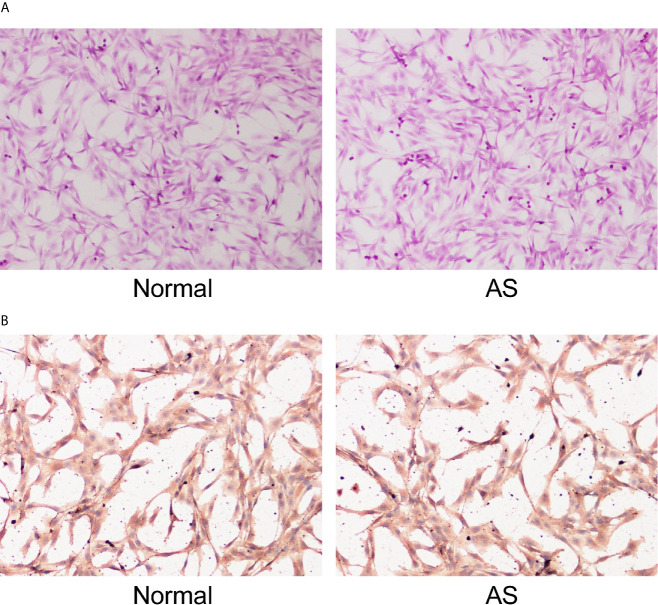
Identification of fibroblasts. Fibroblasts were isolated from AS patients and fracture patients. After osteogenic induction, **(A)** representative images of fibroblasts after osteogenic induction detected by HE staining; **(B)** Representative images of vimentin after osteogenic induction detected by immunocytochemistry. Normal and AS were positive; normal was fibroblasts isolated and cultured from cystic ligament of patients with femoral neck and femoral fracture and as was fibroblasts isolated and cultured from cystic ligament of patients with ankylosing spondylitis.

Fibroblasts obtained from patients with AS and femoral neck fracture were observed and identified with HE staining (**Figure 1A**). Immunocytochemistry of cells was performed with vimentin, a specific marker of fibroblasts (**Figure 1B**). There was no significant difference in morphology between the AS fibroblasts and normal fibroblasts. Cells were in long spindles with oval and regular nuclei. AS fibroblasts and normal fibroblasts were positive for vimentin, confirming that all the cultured cells were fibroblasts from mesoderm.

### miR-214-3p Inhibits AS Fibroblast Osteogenesis

**Figure 2:**
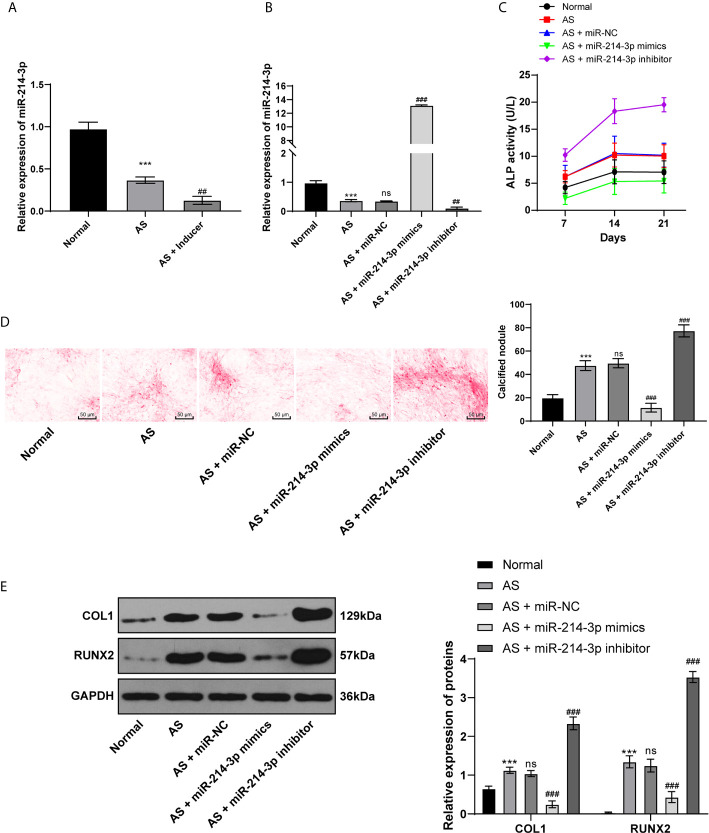
miR-214-3p inhibits AS fibroblast osteogenesis. After osteogenic induction of fibroblasts isolated from AS patients and fracture patients, **(A)** relative miR-214-3p expression was measured by RT-qPCR; then miR-214-3p mimic, inhibitor, and NC were transfected into fibroblasts; **(B)** Relative miR-214-3p expression with different transfections was measured by RT-qPCR; **(C)** Relative ALP activity in AS fibroblasts after osteogenic induction; **(D)** Representative images and histogram of calcified nodule in AS fibroblasts after osteogenic induction detected by ARS staining; **(E)** Protein levels of COL1 and Runx2 in AS fibroblasts after osteogenic induction measured by western blot analysis. Relative to the normal group, ***p < 0.001; *versus* the AS fibroblasts, ^##^p < 0.01, ^###^ *p* < 0.001; ns represents no statistical differences.

miR is a major regulator in many diseases. miR-214 has been reported to be lowly expressed in AS patients and used as a non-invasive biomarker for AS diagnosis (10). Through RT-qPCR, we found that miR-214 expression was lower in AS fibroblasts than in normal fibroblasts before osteogenic induction and further decreased in AS fibroblasts after osteogenic induction (**Figure 2A**). Subsequently, miR-214-3p mimic and miR-214-3p inhibitors were transfected into fibroblasts after induction (**Figure 2B**). ALP activity was measured and the formation of calcium nodules was detected with ARS staining 21 days later. ALP activity of AS fibroblasts was detected at 7^th^, 14^th^, and 21^st^ days after transfection. It was found that ALP activity of AS fibroblasts increased with time after osteogenic induction; miR-214-3p mimic could significantly inhibit ALP activity, while miR-214-3p inhibitor increased ALP activity of AS fibroblasts (**Figure 2C**). As was shown in **Figure 2D**, compared with normal fibroblasts, calcified nodule in AS fibroblasts was markedly increased. miR-214-3p mimic inhibited the formation of calcium nodules in AS fibroblasts, while AS fibroblasts with miR-214-3p inhibitor produced abundant calcium nodules. Additionally, levels of COLI and Runx2 related to osteogenic differentiation were detected in AS fibroblasts (**Figure 2E**). As expected, COLI and Runx2 were increased in AS fibroblasts, but decreased noticeably in AS fibroblasts with miR-214-3p mimic, and reached highest levels in AS fibroblasts with miR-214-3p inhibitor. Runx2 expression was almost not detected in normal fibroblasts. Therefore, we concluded that miR-214-3p could prevent AS fibroblast osteogenesis.

### miR-214-3p Targets BMP2

**Figure 3:**
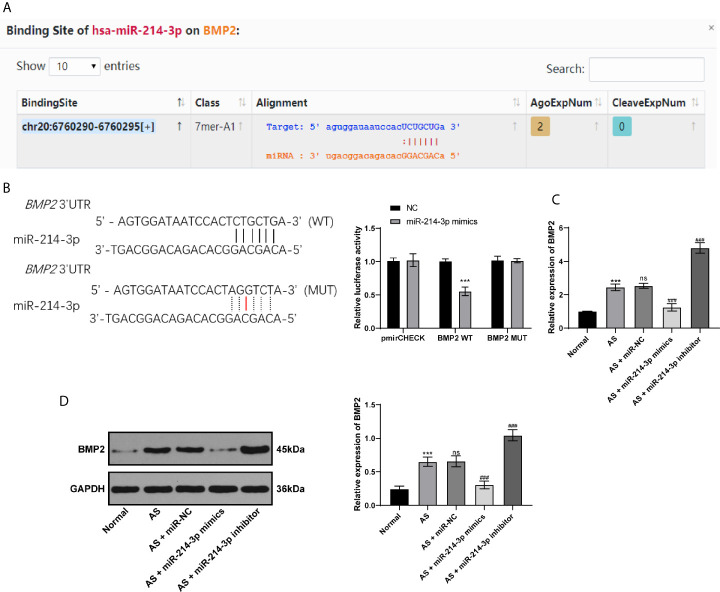
miR-214-3p targets BMP2. **(A)** Binding sites of miR-214-3p on BMP2 *via* bioinformatics analysis and database screening; **(B)** The targeting relationship between miR-214-3p and BMP2 confirmed by dual luciferase reporter gene assay, relative to the NC group, ***p < 0.001; **(C)** mRNA expression of BMP2 measured by RT-qPCR; **(D)** Protein level of BMP2 measured by western blot analysis. Relative to the normal group, ***p < 0.001. ns represents no statistical differences; relative to the AS+miR-NC group, ^###^p < 0.001.

BMP2 is one of the proteins expressed extensively during the development of AS and plays an important role in osteogenesis (21). Through bioinformatics analysis and database screening, we found that there were targeted binding sites between miR-214-3p and BMP2, and the complementary sequence of 3′UTR is shown in **Figure 3A**. In order to confirm the targeting relationship between miR-214-3p and BMP2, we constructed a dual luciferase reporter gene vector and confirmed the targeting relationship between miR-214-3p and BMP2 through luciferase activity (**Figure 3B**). Next, BMP2 levels were measured using RT-qPCR and western blot analysis. The obtained results indicated that BMP2 levels in fibroblasts transfected with miR-214-3p mimic were substantially decreased, which could be reversed by miR-214-3p inhibitor (**Figures 3C, D** **)**. This suggested that BMP2 is indeed the downstream target of miR-214-3p in AS fibroblast osteogenesis and is negatively regulated by miR-214-3p.

### BMP2 Activates the BMP–TGFβ Axis to Promote Osteogenic Differentiation of AS Fibroblasts

**Figure 4:**
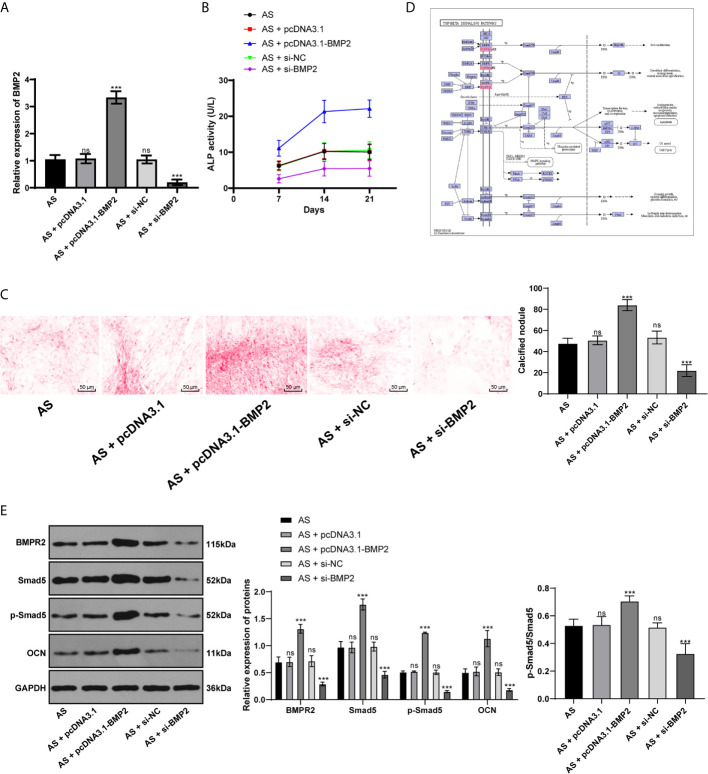
BMP2 promotes osteogenic differentiation of AS fibroblasts by activating BMP–TGF*β* axis. **(A)** Relative BMP2 expression in AS fibroblasts was detected by RT-qPCR; **(B)** Relative ALP activity after transfecting the overexpressing BMP2 plasmid into AS fibroblasts. Overexpression of BMP2 increased ALP activity in AS cells; **(C)** Representative images and histogram of calcified nodule in AS fibroblasts detected by ARS staining. Overexpression of BMP2 increased calcified nodule in AS fibroblasts; **(D)** KEGG pathway diagram; **(E)** Protein levels of downstream proteins in the BMP–TGF*β* axis measured by western blot analysis. Compared with the AS group, ***p < 0.001; ns represents no statistical differences.

Considering BMP2 is indeed the downstream target of miR-214-3p, now we turned to investigate the mechanism of BMP2 in osteogenic differentiation of AS fibroblasts and its potential downstream signaling pathway. Through RT-qPCR and Western blot analysis, we found BMP2 was significantly expressed in AS fibroblasts compared with normal fibroblasts after osteogenic induction. We further transfected the plasmid overexpressing BMP2 or silenced BMP2 into AS fibroblasts (**Figure 4A**). ALP detection (**Figure 4B**) and ARS staining (**Figure 4C**) showed that excessive BMP2 expression promoted AS fibroblast osteogenesis, while silenced BMP2 inhibited AS fibroblast osteogenesis. BMP–TGF*β* is involved in regulating the osteogenic differentiation of AS fibroblasts (22). Through the KEGG signaling pathway, BMPR2 and Smad5 are downstream pathway proteins of BMP2 (**Figure 4D**). Western blot analysis found that BMPR2, p-Smad5, Smad5, p-Smad5/Smad5, and OCN were significantly increased in AS fibroblasts with excessive BMP2 expression, while silenced BMP2 declined the pathway-related factors above (**Figure 4E**), indicating that BMP2 promoted the osteogenic differentiation of AS fibroblasts by activating the BMP–TGF*β* axis.

### miR-214-3p Inhibits BMP2 Expression to Further Repress AS Fibroblast Osteogenesis

**Figure 5:**
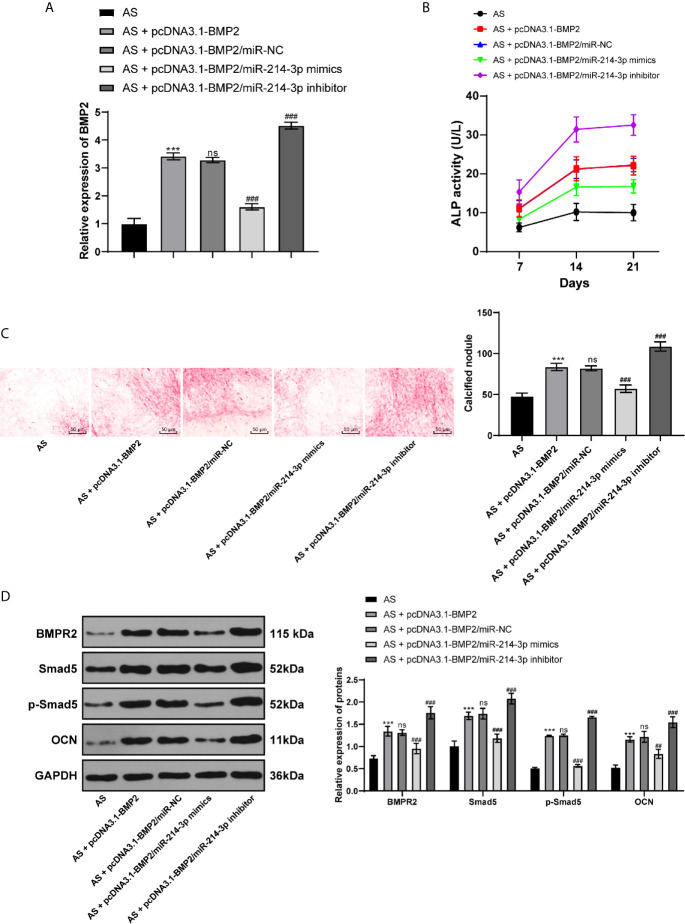
miR-214-3p targets BMP2 and inhibits osteogenic differentiation of AS fibroblasts *via* the BMP–TGF*β* signaling pathway. **(A)** Relative BMP2 expression in AS fibroblasts after different transfection detected by RT-qPCR; **(B)** Relative ALP activity in AS fibroblasts with overexpressing BMP2 and miR-214-3p mimic/inhibitor; **(C)** Representative images and histogram of calcified nodule in AS fibroblasts detected by ARS staining; **(D)** Protein levels of downstream proteins in the BMP–TGFβ axis measured by western blot analysis. Compared with the AS group, ***p < 0.001; compared with the AS + pcDNA3.1-BMP2 group, ^###^ *p* < 0.001; ns represents no statistical differences.

To further confirm the mechanism of miR-214-3p in osteogenic differentiation of AS fibroblasts, miR-214-3p mimic or miR-214-3p inhibitor was transfected into AS fibroblasts with overexpressing BMP2. The results exhibited that the overexpressed BMP2 was inhibited by miR-214-3p mimic, but the highest BMP2 expression was achieved by miR-214-3p inhibitor (**Figure 5A**). Similarly, we recorded ALP activity (**Figure 5B**) and ARS staining (**Figure 5C**) within 21 days. It indicated that miR-214-3p inhibited BMP2 expression and reduced ALP activity and calcium nodule formation. Western blot analysis also found that levels of BMPR2, p-Smad5, Smad5, p-Smad5/Smad5, and OCN were decreased after BMP2 inhibition (**Figure 5D**), suggesting miR-214-3p prevented osteogenic differentiation of AS fibroblasts by targeting BMP2 and inhibiting the BMP–TGF*β* axis.

## Discussion

Although infliximab (a monoclonal antibody against TNF-α) has been commonly shown to be effective in AS treatment, long-term follow-up studies demonstrated the association between infliximab therapy and increased risk of non-Hodgkin’s lymphoma (23). Surprisingly, Md Shaifur Rahman et al. highlighted that BMP plays major roles in regulating osteogenesis and bone formation and implied the clinical applications of TGF*β*–BMP axis for bone diseases (24). It is commonly recognized that miRs have critical roles in regulating TGF*β*–BMP and osteoblast differentiation (25). Based on this, we hypothesized prior to the experiments that miR-214-3p, BMP2, and the TGF*β*/BMP axis may be interacting in the osteogenic differentiation of AS fibroblasts. Collectively, we claimed that miR-214-3p could inhibit osteogenic differentiation of AS fibroblasts by targeting BMP2 and blocking BMP–TGF*β* axis.

The first important observation in this study was that miR-214 expression was lower in AS fibroblasts and further decreased after osteogenic induction, and miR-214-3p overexpression inhibited ALP activity, calcified nodules, COLI, and Runx2 levels. Consistently, miR-214-3p was downregulated in osteogenic differentiation of MSMSCs, and miR-214-3p overexpression substantially diminished levels of osteogenic markers Runx2 and ALP in osteogenic differentiated MSMSCs (11). As recently reported, upregulated osteoclastic miR-214-3p led to inhibited osteoblast activity and bone formation in aged women with fractures (26). Runx2 is a major regulator of osteogenic differentiation by regulating key osteogenic genes, and Runx2 knockout in mice results in complete depletion of bone formation (27). Runx2 and ALP are early markers of osteogenesis; OCN is a late osteogenic marker, and calcified nodules are signs of the final stage of osteogenesis (28, 29). Kihara T previously indicated that exogenous COLI increased calcified nodules, osteogenesis, and matrix mineralization of mesenchymal stem cells (30). Importantly, enhanced expression of ALP and Runx2 induced osteogenic differentiation, which stimulated AS progression (31). Interestingly, miR-214 level was positively associated with the number of white blood cells, platelets, C-reactive protein (CRP), and AS Disease Activity Score with CRP (10). In summary, miR-214-3p could prevent osteogenic differentiation of AS fibroblasts.

Furthermore, the data confirmed that BMP2 is a target gene of miR-214-3p. As Ramazzotti G et al. demonstrated, overexpressed miR-214 inhibited osteogenic differentiation of C2C12 cells by suppressing phosphoinositide-phospholipase C*β*1, which was crucial to promote BMP-2-induced osteogenesis (32). BMPs could exert potent function in regulating osteoblastic stem cells, among which recombinant human BMP2 is applicable in clinical adjuvant therapy for bone formation (33). Besides, we also found overexpressing BMP2 increased levels of BMPR2, Smad5, p-Smad5, and OCN in AS fibroblasts, activated BMP–TGF*β* signaling pathway, and promoted osteogenic differentiation of AS fibroblasts. Miron RJ et al. demonstrated that administration of BMP2 increased levels of osteoblast differentiation markers COLI and OCN and promoted mineralization of primary osteoblasts (16). After BMP activation, Runx2 and Smad interacted to regulate the transcription of target genes, and then induced osteoblast differentiation of mesenchymal precursor cells (24). Shea Carter et al. noted that blockade of BMP signaling resulted in protection against arthritis and ankylosis, which was beneficial for AS prevention and treatment (14). Moreover, our data highlighted that miR-214-3p could reverse the promoting effects of BMP2 on osteogenic differentiation of AS fibroblasts. A recent research revealed that miRs regulated osteogenic differentiation *via* osteoblast-related genes, particularly Runx2 and BMP2 and *via* signaling pathways like TGF*β* and BMPs (34). BMPs and TGF*β* could facilitate osteoblast proliferation and differentiation *in vitro* and speed up new bone formation *in vivo* (16). Strikingly, evidence revealed that TGF*β* could induce fibroblast synthesis of COLI, OCN, and ALP, block extracellular matrix digestion, promote the amount of bone and cartilage tissues and bone formation in AS (22). Similarly, miR-214-5p overexpression diminished levels of ALP, Runx2, OCN and COLI, and TGF*β*/Smad2 in bone marrow stem cells of postmenopausal osteoporosis (18). Taken together, the inhibitory effects of miR-214-3p on osteogenic differentiation of AS fibroblasts were achieved *via* BMP2 and the BMP–TGF*β* axis. However, the change of Smad5 protein itself may be caused by other target genes downstream of miR-214-3p, which will be further studied in the future.

To sum up, our study supported the notion that miR-214-3p could inhibit osteogenic differentiation of AS fibroblasts by targeting BMP2 and blocking the BMP–TGF*β* axis, highlighting promising novel approaches for AS treatment. There are many studies on osteoblast differentiation in normal medium (35, 36). On the basis of these studies, we will carry out relevant research on whether miR-214-3p can promote osteogenesis in normal medium in the future. More studies are still needed to further validate our results and deeply analyze the molecular mechanism of AS so as to find out applicable methods for AS patients in the clinic.

## Data Availability Statement

The original contributions presented in the study are included in the article/supplementary material. Further inquiries can be directed to the corresponding authors.

## Ethics Statement

The studies involving human participants were reviewed and approved by the Clinical Ethical Committee of Beijing Shijitan Hospital. The patients/participants provided their written informed consent to participate in this study. The animal study was reviewed and approved by Clinical Ethical Committee of Beijing Shijitan Hospital.

## Author Contributions

YY contributed to the study design and manuscript preparation. LD contributed to the definition of intellectual content and manuscript editing. YH is the guarantor of integrity of the entire study. HJ took charge of the literature research. JZ contributed to experimental studies. ZD contributed to the acquisition and analysis of data. GZ contributed to the study concepts. All authors contributed to the article and approved the submitted version.

## Funding

This study was supported by the National Natural Science Foundation of China (81904215) and Beijing Traditional Chinese Medicine Science and Technology Development Fund (2018-A29), Capital Clinical Application Research and Achievement Promotion (Z151100004015068).

## Conflict of Interest

The authors declare that the research was conducted in the absence of any commercial or financial relationships that could be construed as a potential conflict of interest.

## References

[1] Evans DM Spencer CC Pointon JJ Su Z Harvey D Kochan G. Interaction between ERAP1 and HLA-B27 in ankylosing spondylitis implicates peptide handling in the mechanism for HLA-B27 in disease susceptibility. Nat Genet (2011) 43:761–7. 10.1038/ng.873 PMC3640413 21743469

[2] Baeten D Baraliakos X Braun J Sieper J Emery P van der Heijde D. Anti-interleukin-17A monoclonal antibody secukinumab in treatment of ankylosing spondylitis: a randomised, double-blind, placebo-controlled trial. Lancet 382:1705–13. 10.1016/S0140-6736(13)61134-4 24035250

[3] Law L Beckman Rehnman J Deminger A Klingberg E Jacobsson LTH Forsblad-d’Elia H. Factors related to health-related quality of life in ankylosing spondylitis, overall and stratified by sex. Arthritis Res Ther 20:284. 10.1186/s13075-018-1784-8 PMC6307231 30587228

[4] Min Y Hui-Yun G Hou-Cheng Z Yuan-Long X Wei J Lin C. The surgical treatment strategies for thoracolumbar spine fractures with ankylosing spondylitis: a case report. BMC Surg 19:99. 10.1186/s12893-019-0565-x PMC6660961 31349822

[5] Schett G Rudwaleit M. Can we stop progression of ankylosing spondylitis? Best Pract Res Clin Rheumatol 24:363–71. 10.1016/j.berh.2010.01.005 20534370

[6] Mathieu S Gossec L Dougados M Soubrier M. Cardiovascular profile in ankylosing spondylitis: a systematic review and meta-analysis. Arthritis Care Res (Hoboken) 63:557–63. 10.1002/acr.20364 20890982

[7] Isogai N Asamoto S Nakamura S Sakurai K Ishihara S Ishikawa M. Spine and Spinal Cord Injury Associated with a Fracture in Elderly Patients with Ankylosing Spondylitis. Neurol Med Chir (Tokyo) 58:103–9. 10.2176/nmc.oa.2017-0112 PMC5929918 29269632

[8] Chou CH Shrestha S Yang CD Chang NW Lin YL Liao KW. miRTarBase update 2018: a resource for experimentally validated microRNA-target interactions. Nucleic Acids Res 46:D296–302. 10.1093/nar/gkx1067 PMC5753222 29126174

[9] Lai NS Yu HC Chen HC Yu CL Huang HB Lu MC. Aberrant expression of microRNAs in T cells from patients with ankylosing spondylitis contributes to the immunopathogenesis. Clin Exp Immunol 173:47–57. 10.1111/cei.12089 PMC3694534 23607629

[10] Kook HY Jin SH Park PR Lee SJ Shin HJ Kim TJ. Serum miR-214 as a novel biomarker for ankylosing spondylitis. Int J Rheum Dis 22:1196–201. 10.1111/1756-185X.13475 30729703

[11] Peng W Zhu S Chen J Wang J Rong Q Chen S. Hsa_circRNA_33287 promotes the osteogenic differentiation of maxillary sinus membrane stem cells via miR-214-3p/Runx3. BioMed Pharmacother 109:1709–17. 10.1016/j.biopha.2018.10.159 30551425

[12] Xie Z Wang P Li Y Deng W Zhang X Su H. Imbalance Between Bone Morphogenetic Protein 2 and Noggin Induces Abnormal Osteogenic Differentiation of Mesenchymal Stem Cells in Ankylosing Spondylitis. Arthritis Rheumatol 68:430–40. 10.1002/art.39433 26413886

[13] Lories RJ Derese I Luyten FP. Modulation of bone morphogenetic protein signaling inhibits the onset and progression of ankylosing enthesitis. J Clin Invest 115:1571–9. 10.1172/JCI23738 PMC1090472 15902307

[14] Carter S Braem K Lories RJ. The role of bone morphogenetic proteins in ankylosing spondylitis. Ther Adv Musculoskelet Dis 4:293–9. 10.1177/1759720X12444175 PMC3403253 22859928

[15] Yang C Ding P Wang Q Zhang L Zhang X Zhao J. Inhibition of Complement Retards Ankylosing Spondylitis Progression. Sci Rep 6:34643. 10.1038/srep34643 PMC5048143 27698377

[16] Miron RJ Saulacic N Buser D Iizuka T Sculean A. Osteoblast proliferation and differentiation on a barrier membrane in combination with BMP2 and TGFbeta1. Clin Oral Investig 17:981–8. 10.1007/s00784-012-0764-7 22669486

[17] Chen G Deng C Li YP. TGF-beta and BMP signaling in osteoblast differentiation and bone formation. Int J Biol Sci 8:272–88. 10.7150/ijbs.2929 PMC3269610 22298955

[18] Qiu J Huang G Na N Chen L. MicroRNA-214-5p/TGF-beta/Smad2 signaling alters adipogenic differentiation of bone marrow stem cells in postmenopausal osteoporosis. Mol Med Rep 17:6301–10. 10.3892/mmr.2018.8713 PMC5928609 29532880

[19] Schmittgen TD Livak KJ. Analyzing real-time PCR data by the comparative C(T) method. Nat Protoc 3:1101–8. 10.1038/nprot.2008.73 18546601

[20] Li JH Liu S Zhou H Qu LH Yang JH. starBase v2.0: decoding miRNA-ceRNA, miRNA-ncRNA and protein-RNA interaction networks from large-scale CLIP-Seq data. Nucleic Acids Res 42:D92–7. 10.1093/nar/gkt1248 PMC3964941 24297251

[21] Zheng G Xie Z Wang P Li J Li M Cen S. Enhanced osteogenic differentiation of mesenchymal stem cells in ankylosing spondylitis: a study based on a three-dimensional biomimetic environment. Cell Death Dis 10:350. 10.1038/s41419-019-1586-1 PMC6484086 31024000

[22] Jia C Liu H Li M Wu Z Feng X. Effects of icariin on cytokine-induced ankylosing spondylitis with fibroblastic osteogenesis and its molecular mechanism. Int J Clin Exp Pathol 7:9104–9. PMC4314045 25674296

[23] Dauendorffer JN Rivet J Allard A Bachelez H. Sezary syndrome in a patient receiving infliximab for ankylosing spondylitis. Br J Dermatol 156:742–3. 10.1111/j.1365-2133.2006.07713.x 17263820

[24] Rahman MS Akhtar N Jamil HM Banik RS Asaduzzaman SM. TGF-beta/BMP signaling and other molecular events: regulation of osteoblastogenesis and bone formation. Bone Res 3:15005. 10.1038/boneres.2015.5 PMC4472151 26273537

[25] Grunhagen J Bhushan R Degenkolbe E Jager M Knaus P Mundlos S. MiR-497 approximately 195 cluster microRNAs regulate osteoblast differentiation by targeting BMP signaling. J Bone Miner Res 30:796–808. 10.1002/jbmr.2412 25407900

[26] Li D Liu J Guo B Liang C Dang L Lu C. Osteoclast-derived exosomal miR-214-3p inhibits osteoblastic bone formation. Nat Commun 7:10872. 10.1038/ncomms10872 PMC4786676 26947250

[27] Kidwai F Edwards J Zou L Kaufman DS. Fibrinogen Induces RUNX2 Activity and Osteogenic Development from Human Pluripotent Stem Cells. Stem Cells 34:2079–89. 10.1002/stem.2427 PMC5097445 27331788

[28] Vaes BL Lute C van der Woning SP Piek E Vermeer J Blom HJ. Inhibition of methylation decreases osteoblast differentiation via a non-DNA-dependent methylation mechanism. Bone 46:514–23. 10.1016/j.bone.2009.09.033 19815105

[29] Liu Y Lin Z Guo J Xu G Li Y Xu T. Notoginsenoside R1 significantly promotes in vitro osteoblastogenesis. Int J Mol Med 38:537–44. 10.3892/ijmm.2016.2652 27352906

[30] Kihara T Hirose M Oshima A Ohgushi H. Exogenous type I collagen facilitates osteogenic differentiation and acts as a substrate for mineralization of rat marrow mesenchymal stem cells in vitro. Biochem Biophys Res Commun 341:1029–35. 10.1016/j.bbrc.2006.01.059 16458256

[31] Osta B Lavocat F Eljaafari A Miossec P. Effects of Interleukin-17A on Osteogenic Differentiation of Isolated Human Mesenchymal Stem Cells. Front Immunol 5:425. 10.3389/fimmu.2014.00425 25228904 PMC4151036

[32] Ramazzotti G Bavelloni A Blalock W Piazzi M Cocco L Faenza I. BMP-2 Induced Expression of PLCbeta1 That is a Positive Regulator of Osteoblast Differentiation. J Cell Physiol 231:623–9. 10.1002/jcp.25107 26217938

[33] Ye G Li C Xiang X Chen C Zhang R Yang X. Bone morphogenetic protein-9 induces PDLSCs osteogenic differentiation through the ERK and p38 signal pathways. Int J Med Sci 11:1065–72. 10.7150/ijms.8473 PMC4135228 25136261

[34] Huang C Geng J Jiang S. MicroRNAs in regulation of osteogenic differentiation of mesenchymal stem cells. Cell Tissue Res (2017) 368:229–38. 10.1007/s00441-016-2462-2 27425852

[35] Maung WM Nakata H Miura M Miyasaka M Kim YK Kasugai S. Low-Intensity Pulsed Ultrasound Stimulates Osteogenic Differentiation of Periosteal Cells In Vitro. Tissue Eng Part A (2020) 27:63–73. 10.1089/ten.TEA.2019.0331 32164486

[36] Veernala I Giri J Pradhan A Polley P Singh R Yadava SK. Effect of Fluoride Doping in Laponite Nanoplatelets on Osteogenic Differentiation of Human Dental Follicle Stem Cells (hDFSCs). Sci Rep (2019) 9:1223–37. 10.1038/s41598-018-37327-7 PMC6351553 30696860

